# AAV9-mediated gene delivery to liver grafts during static cold storage in a rat liver transplant model

**DOI:** 10.3389/frtra.2023.1171272

**Published:** 2023-05-30

**Authors:** Qimeng Gao, Samuel J. Kesseli, Trevor Gonzalez, Min Zhang, Riley Kahan, Madison Krischak, Samantha E. Halpern, Mingqing Song, Hongzhi Xu, Nader Abraham, Imran J. Anwar, Isaac Alderete, Aravind Asokan, Matthew G. Hartwig, Andrew S. Barbas

**Affiliations:** ^1^ Department of Surgery, Duke University Medical Center, Durham, NC, United States; ^2^ Department of Molecular Genetics & Microbiology, Duke University School of Medicine, Durham, NC, United States; ^3^ Department of Pathology, Duke University Medical Center, Durham, NC, United States; ^4^ Department of Biomedical Engineering, Duke University, Durham, NC, United States; ^5^ Division of Cardiothoracic Surgery, Department of Surgery, Duke University Medical Center, Durham, NC, United States

**Keywords:** adeno-associated virus, rat, liver transplant, static cold storage, gene therapy

## Abstract

**Introduction:**

Recombinant adeno-associated virus (rAAV) is a novel strategy used clinically for gene delivery, but has not been characterized in the context of organ transplantation. We sought to determine the efficacy of rAAV-mediated gene delivery during static cold storage (SCS) prior to liver transplantation.

**Methods:**

A triple-plasmid transfection protocol was used to produce rAAV subtype-9 vectors containing firefly luciferase genomes in HEK293 cells. Lewis rat liver grafts were flushed and stored in cold HTK solution. Three experimental groups received rAAV at different doses, administered via the portal vein as a bolus during SCS. A control group did not receive rAAV (*N* = 2). Recipients then underwent syngeneic liver transplantation. Bioluminescence imaging to quantify *in vivo* luciferase expression was performed on post-operative days 7, 14, 28, and 56.

**Results:**

Control animals demonstrated no bioluminescent activity, while animals receiving rAAV-treated livers had increasing bioluminescence, peaking at four weeks but sustained to the eight-week endpoint. This result was confirmed by experimental endpoint tissue luciferase activity assay.

**Discussion:**

rAAV mediates gene transduction in liver grafts when administered during SCS and has potential for gene therapy applications in solid organ transplantation.

## Introduction

Solid organ transplantation remains limited by the shortage of suitable donor organs and suboptimal graft longevity due to chronic immunologic injury. As such, novel approaches for improving the function of high-risk grafts and reducing graft immunogenicity have become necessary.

Gene therapy has emerged as a powerful tool with potential to address these challenges in transplantation. Historically intended for the treatment of inherited monogenic disorders, the term “gene therapy” now more broadly refers to any genetic modification of cells to produce a therapeutic effect, through promotion, modification, or silencing of target protein expression. A critical aspect of successful gene therapy is organ-specific delivery, a process facilitated by the sequence of events inherent to solid organ transplantation. Donor organs are procured and undergo a period of preservation, providing a window during which therapies can be delivered directly to the organ of interest with limited off-target effects.

Early studies examining gene delivery to an isolated donor organ focused on recombinant adenovirus (1–3). However, the effect of adenoviral-mediated gene transfer tends to be short-lived, with a quick onset and offset within days. In addition, strong innate responses against adenoviral capsid proteins have the potential to trigger a lethal cytokine storm (4). More recently, adeno-associated viral (AAV) vectors have emerged as the leading platform for therapeutic gene delivery for a variety of human diseases, with AAV-based products obtaining approval from the U.S. Food and Drug Administration (FDA) in recent years (5). AAV vectors naturally target the liver and AAV infection causes no known disease in humans. Thus far, the use of AAV-mediated gene transfer has not been well studied in the context of solid organ transplantation.

Herein, we report a proof-of-concept study evaluating the feasibility of *ex vivo* gene delivery to liver grafts using an AAV9 vector administered during static cold storage (SCS). We demonstrate sustained transgene expression in transplanted liver grafts out to 8 weeks with limited off-target effects, suggesting that AAV-mediated gene delivery represents a feasible approach for long-term graft modulation.

## Materials and methods

### Animals

Male Lewis rats (Charles River Laboratory, Wilmington, MA, USA) weighing 200–250 g were used in the study. All protocols were approved by Duke University Institutional Animal Care and Use Committee. Animals were housed in standard pathogen-free conditions in accordance with the “Guide for the Care and Use of Laboratory Animals” published by the National Institutes of Health (8th edition, 2011).

### Experimental design

**Figure 1:**
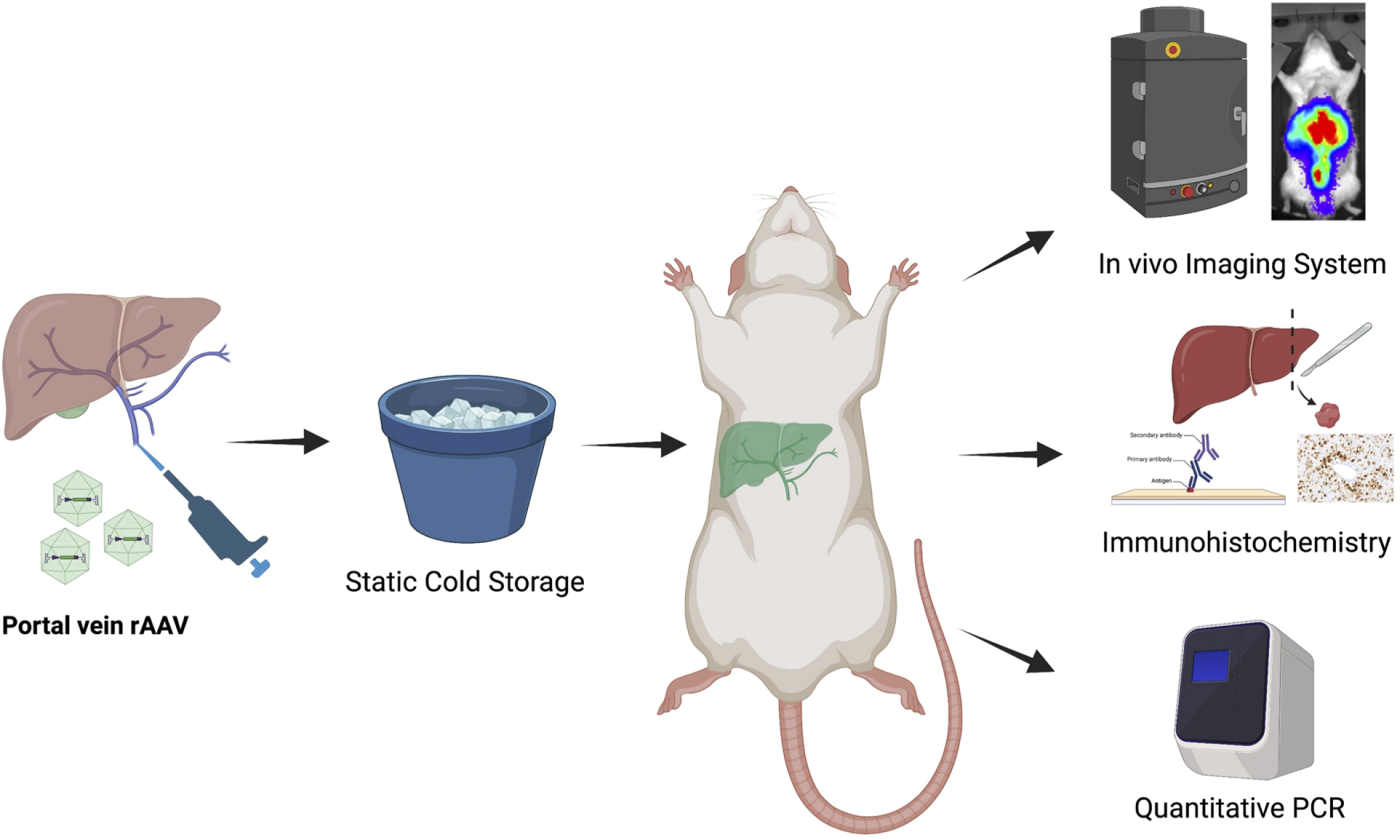
Overall experimental design of the study. Livers from Lewis rat donors were procured and then preserved on ice, following a bolus injection of solution containing recombinant AAV9-Luc (low dose: 8e10vg, *n* = 4; intermediate dose: 2e11vg, *n* = 4; high dose: 4e11vg, *n* = 8). Control animals received no AAV9-Luc. A separate Lewis rat was used as recipient of the liver transplant. Following orthotopic liver transplantation, all recipients were followed for 2 months. *In vivo* bioluminescence imaging was performed at posttransplant day 7, 14, 28, and 56 to monitor luciferase transgene expression. At the end of study period, animals were euthanized. Tissues were collected for viral genome biodistribution analysis and *in vitro* luciferase activity assay.

Lewis rat liver grafts were procured and subjected to SCS before transplantation (Figure 1). In the control group, grafts were flushed with and maintained in cold (4°C) Belzer UW cold storage solution or Custodiol HTK solution before implantation. In the experimental groups, recombinant AAV9 encoding a single-stranded firefly luciferase reporter was administered through the portal vein at the start of SCS. At the end of the 30-min preservation period, a syngeneic orthotopic liver transplant was performed using the preserved isograft. A 30-min interval was chosen as a donor hepatectomy generally takes 30 min and previous studies have shown that vector-mediated gene transduction can be achieved within such a time frame (6). All recipients were followed for 2 months, during which animals underwent *in vivo* bioluminescence measurement on posttransplant days 7, 14, 28, and 56 to monitor transgene expression. On day 56, the animals were euthanized and tissues were collected for immunohistochemistry, luciferase activity assay, and quantitative PCR (qPCR). One animal received a liver graft treated with recombinant AAV9 encoding a self-complementary green fluorescence protein (GFP) via the portal vein and was sacrificed on posttransplant day 5 to evaluate the onset of transgene expression when the AAV9 vector delivered was self-complementary in design.

### Experimental procedure

The donor procedure was performed as described previously (7). After procurement, vascular cuffs were placed in the donor infrahepatic inferior vena cava and portal vein. The solution containing AAV9 vectors was delivered into the portal vein using a pipette. A microsurgery vascular clip was then applied to the portal vein to prevent spillage of the AAV vector. The donor organ was then placed on ice during recipient native hepatectomy. An orthotopic liver transplant was performed using a modified vascular cuff technique previously described (8). The suprahepatic caval anastomosis was performed with a running 8–0 suture, while both infrahepatic vena cava and portal vein anastomoses were completed using cuffs. The bile duct anastomosis was performed by placing a stent between the donor and recipient bile duct. No hepatic arterial anastomosis was performed. Anhepatic time, defined by the time from recipient hepatectomy to restoration of blood flow through the portal vein and suprahepatic vena cava, was generally less than 20 min.

### Vector design and production

An AAV9 vector was chosen for its demonstrated tissue tropism to liver. The AAV9 vector (AAV9-Luc) used is single-stranded and contains firefly luciferase with a chimeric CMV-chicken β-actin (CBA) promoter. A triple-plasmid transfection protocol was used to produce recombinant AAV vectors in adherent HEK293 cells. Specifically, the transfected plasmids include a capsid-specific helper plasmid (containing AAV2 Rep and AAV9 Cap genes), the adenoviral helper plasmid pXX680, and pTR-CBA-Luciferase plasmid, flanked by inverted terminal repeats derived from the AAV2 genome. Culture media was harvested on days 4 and 6 after transfection. After the day 4 harvest, fresh Dulbecco's modified eagle medium (DMEM) culture media was added to the cells. After the day 6 media harvest, viral particles were precipitated from the media overnight at 4°C with polyethylene glycol (PEG). The resulting PEG pellet was then resuspended in formulation buffer (1 × phosphate-buffered saline (PBS) with 1 mM MgCl and 0.001% puronic F-68) and treated with DNase at 37°C for 1 h. Vectors were purified using iodixanol density gradient ultracentrifugation and subjected to buffer exchange/desalting using Zeba Spin desalting columns (40,000 MWCO, Thermo Scientific, catalog no. 87770). After purification, viral genome titers were determined via qPCR using a Roche Lightcycler 480 (Roche Applied Sciences). Quantitative PCR primers were designed to specifically recognize the AAV2 inverted terminal repeats (forward, 5′-AACATGCTACGCAGAGAGGGAGTGG-3′; reverse, 5′-CATGAGACAAGGAACCCCTAGTGATGGAG-3′) (Integrated DNA Technologies).

### *In vivo* bioluminescence imaging

*In vivo* bioluminescence imaging was utilized throughout the study period to evaluate luciferase transgene expression. On postoperative days 7, 14, 28, and 56, recipient rats were shaved from the chest to the abdomen under sedation to decrease interference and artifact. A total of 30-mg D-Luciferin was dissolved in 1-ml normal saline solution and injected subcutaneously to the animal, at least 15 min before imaging. All imaging was acquired using the IVIS Kinetic (PerkinElmer, Boston, MA, USA) bioluminescence imaging system using an open filter while the animals were secured in the imaging chamber in the supine position under anesthesia.

### Luciferase assay

Heart, lung, liver, kidney, and spleen tissues were collected at the experimental endpoint. Preweighed rat tissue (0.020–0.040 g) was then incubated in 1 × passive lysis buffer (Promega) following metal bead agitation using a Tissue homogenizer (QIAGEN) at room temperature. The resulting supernatant was collected as the tissue lysate, which is then incubated with luciferin (Promega) for 2 min. Luciferase activity was measured with a Victor ×3 plate reader (PerkinElmer). Relative light units (RLU) for each sample were normalized to tissue mass (g).

### Determination of vector biodistribution by qPCR

DNA was extracted from the tissue using a Purelink Genomic DNA Mini kit (Thermo Fisher Scientific) following the manufacturer’s instructions. Vector genomes were quantified via qPCR, using a plasmid standard and primers targeting SV40 polyA. The biodistribution of viral genomes is represented as the ratio of vector genomes per microgram of DNA extracted.

### Immunohistochemistry

Transplanted liver graft tissues were fixed in 10% formalin and embedded in paraffin. Paraffin sections were stained with hematoxylin and eosin for morphologic observation and luciferase immunohistochemistry. Whole slide digital images were captured using the Aperio AT Turbo digital slide scanner system (Leica Biosystems, Vista, CA, USA) and viewed with Imagescope (Leica Biosystems) digital pathology software.

### Statistical analysis

GraphPad Prism Software (GraphPad Software Inc., La Jolla, CA, USA) was used for the statistical analysis. All values are presented as mean ± SD. *P* < 0.05 is defined as statistical significance. The Mann–Whitney or Kruskal–Wallis test was applied to compare continuous variables between groups.

## Results

### AAV-mediated reporter gene expression can be achieved when delivered during static cold storage

**Figure 2:**
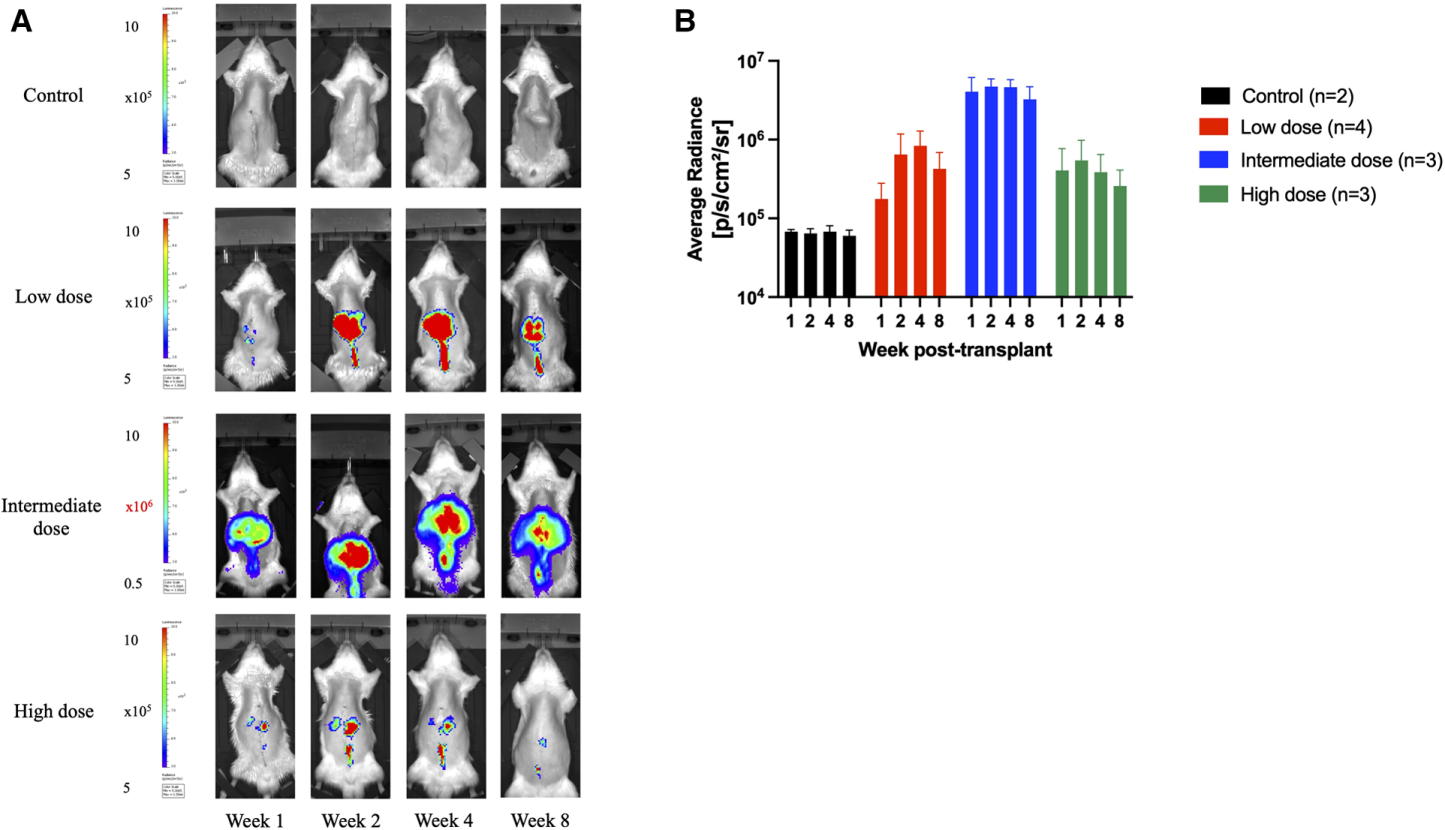
Longitudinal transgene expression as measured by bioluminescence signals. (**A**) Longitudinal bioluminescent images at 1, 2, 4, and 8 weeks following orthotopic liver transplantation of one representative animal from each group that received no vector (control) or AAV9-Luc via portal vein at low dose (8e10 vg), intermediate dose (2e11vg), and high dose (4e11 vg). Signal concentrates in the right upper abdomen in all animals. Note color scale is 1.5–5 x 10^5^ photons per second per square centimeter per steradian (p/s/cm^2^/sr) for all images except the 2e11 group, which required a higher scale of 0.5–10 × 10^6^ p/s/cm^2^/sr. (**B**) Mean radiance over time for each group in p/s/cm^2^/sr (control: black, low dose: red, intermediate dose: blue, high dose: green). Animals that received liver grafts treated with the intermediate AAV9-Luc doses had a higher bioluminescence signal than the low-dose group. *Y*-axis is logarithmic in scale. Data displayed as mean with error bar representing standard deviation.

In total, 18 syngeneic rat liver transplants were performed in the study. Two donor grafts received no AAV9-Luc, four received 8e10 viral genomes (vg) (low-dose group), four received 2e11vg (intermediate dose group), and eight received 4e11vg (high-dose group). All animals that received AAV9-Luc, regardless of dosing, had detectable levels of bioluminescence signals at some point during the 56-day follow-up (Figures 2A, B). For all except one animal, luciferase expression was detected by day 7 and persisted throughout the 2-month study period (Supplemental Figure S1).

### Expression of transgene is increased when higher concentrations of AAV9-Luc are delivered, but appears to be limited by toxicity

Animals that received the intermediate dose demonstrated higher bioluminescence signals than those that received the low dose (intermediate: 4.1e6 p/s/cm^2^/sr vs low: 1.8e5 p/s/cm^2^/sr at week 1, *P* = 0.057; intermediate: 4.7e6 p/s/cm^2^/sr vs low: 6.5e5 p/s/cm^2^/sr at week 2, *P* = 0.057; intermediate: 4.7e6 p/s/cm^2^/sr vs low: 8.3e5 p/s/cm^2^/sr at week 4, *P* = 0.057; intermediate: 3.3e6 p/s/cm^2^/sr vs low: 1.8e5 p/s/cm^2^/sr at week 8, *P* = 0.057). Both groups had more signal detected on *in vivo* imaging at all timepoints than the control animals (mean background average radiance: 6.5e4 p/s/cm^2^/sr).

**Figure 3:**
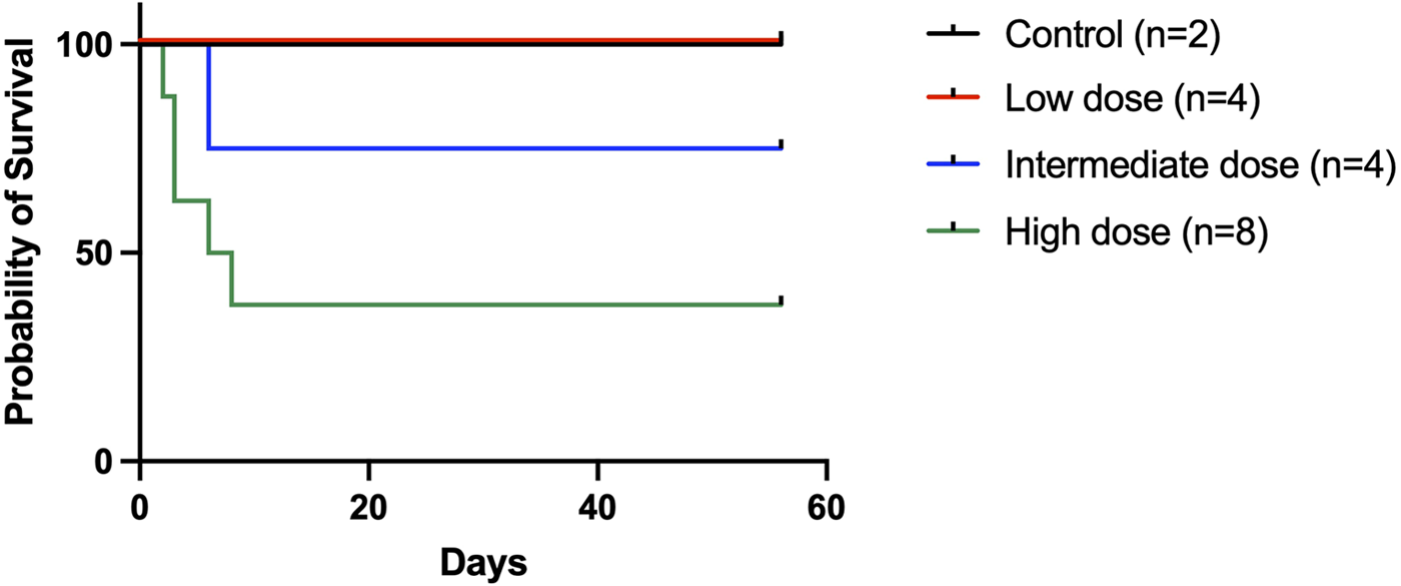
Recipient survival following an orthotopic liver transplant. Recipient survival was 100% in the low-dose group, 75% in the intermediate dose group, and 37.5% in the high-dose group. The five deaths in the high-dose group occurred on posttransplant days 2, 3, 3, 6, and 8, respectively. Three animals were available for postmortem examination (one in the intermediate dose group and two in the high-dose group) and all had significant ascites present.

All animals in the control and low-dose groups survived to the 2-month experimental endpoint. Of the four animals that received the intermediate dose, 3 (75%) survived to the study endpoint, while of the eight animals that received the high dose, only 3 (37.5%) survived to the endpoint (Figure 3).

Interestingly, bioluminescence signals were comparable between animals that received the low dose and the three surviving animals in the high-dose group (high dose: 5.5e5 p/s/cm^2^/sr at week 2; 3.9e5 p/s/cm^2^/sr at week 4; 2.6e5 p/s/cm^2^/sr at week 8). We retrospectively examined the AAV delivery technique in the high-dose group and found that the three animals that survived to the endpoint did not have the graft portal vein clamped after delivery, possibly leading to a leakage of vector in those three cases and a lower effective dose. The rest of the animals in the high-dose group received donor liver grafts that had the portal vein clamped after the AAV9-Luc infusion, and died on days 2, 3, 3, 6, and 8, respectively. Two animals in the group were available for necropsy, which revealed significant ascites.

### Transgene expression is confirmed at the experimental endpoint

**Figure 4:**
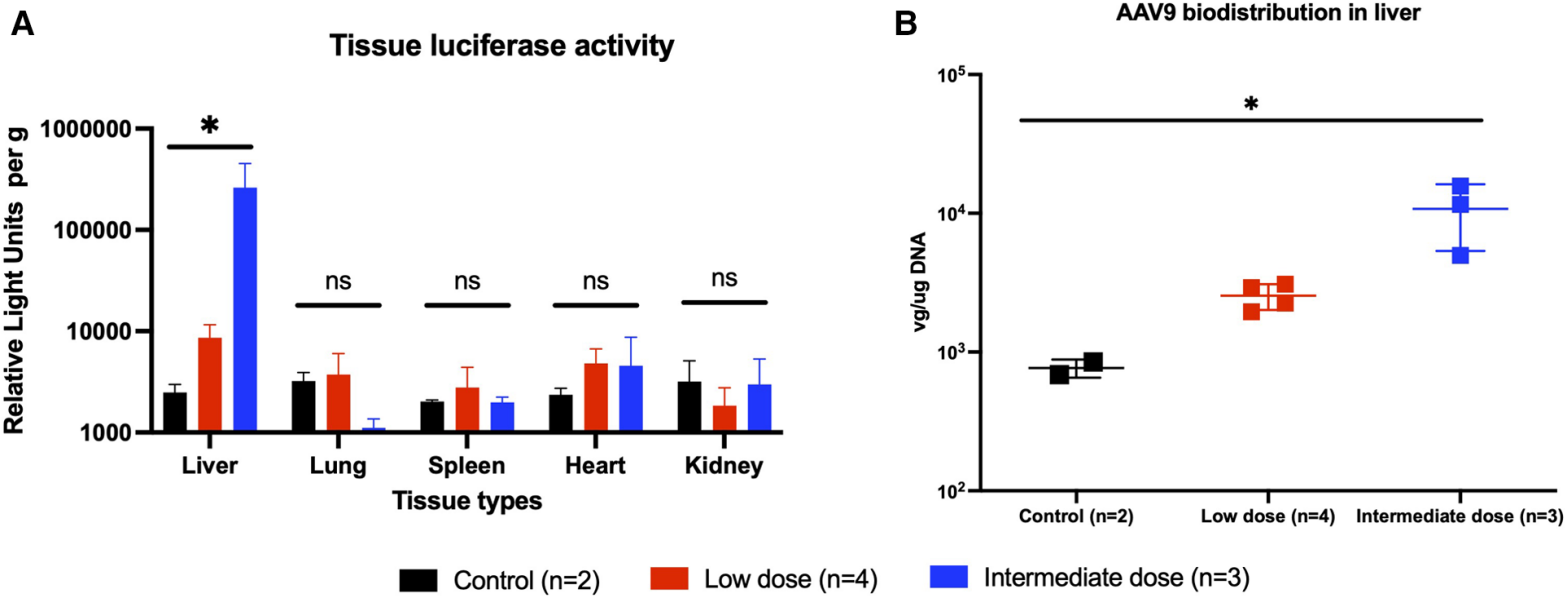
Endpoint transgene expression across different tissue types as measured by tissue luminescence and AAV9-Luc biodistribution in liver as measured by quantitative PCR. (**A**) Animals were sacrificed on posttransplant day 56 (week 8). Liver, lung, spleen, heart, and kidney tissues were collected and analyzed for luciferase activity. Individual tissue luminescence in RLU per gram tissue is shown here. Transplanted liver tissues from the intermediate dose group (blue) demonstrated more luciferase activity than those from the low-dose group (red), and both were above baseline (*P* = 0.0048). There were no differences in luciferase activity in other tissues across all groups, suggesting minimal off-target transgene expression. (**B**) DNA was extracted from liver tissue and qPCR was performed to examine biodistribution of AAV9-Luc following delivery. Transplanted liver tissue from the intermediate dose group had more AAV9-Luc viral copies per µg DNA than the low-dose group (*p* = 0.0048). * denotes *p* < 0.05. ns, not significant.

**Figure 5:**
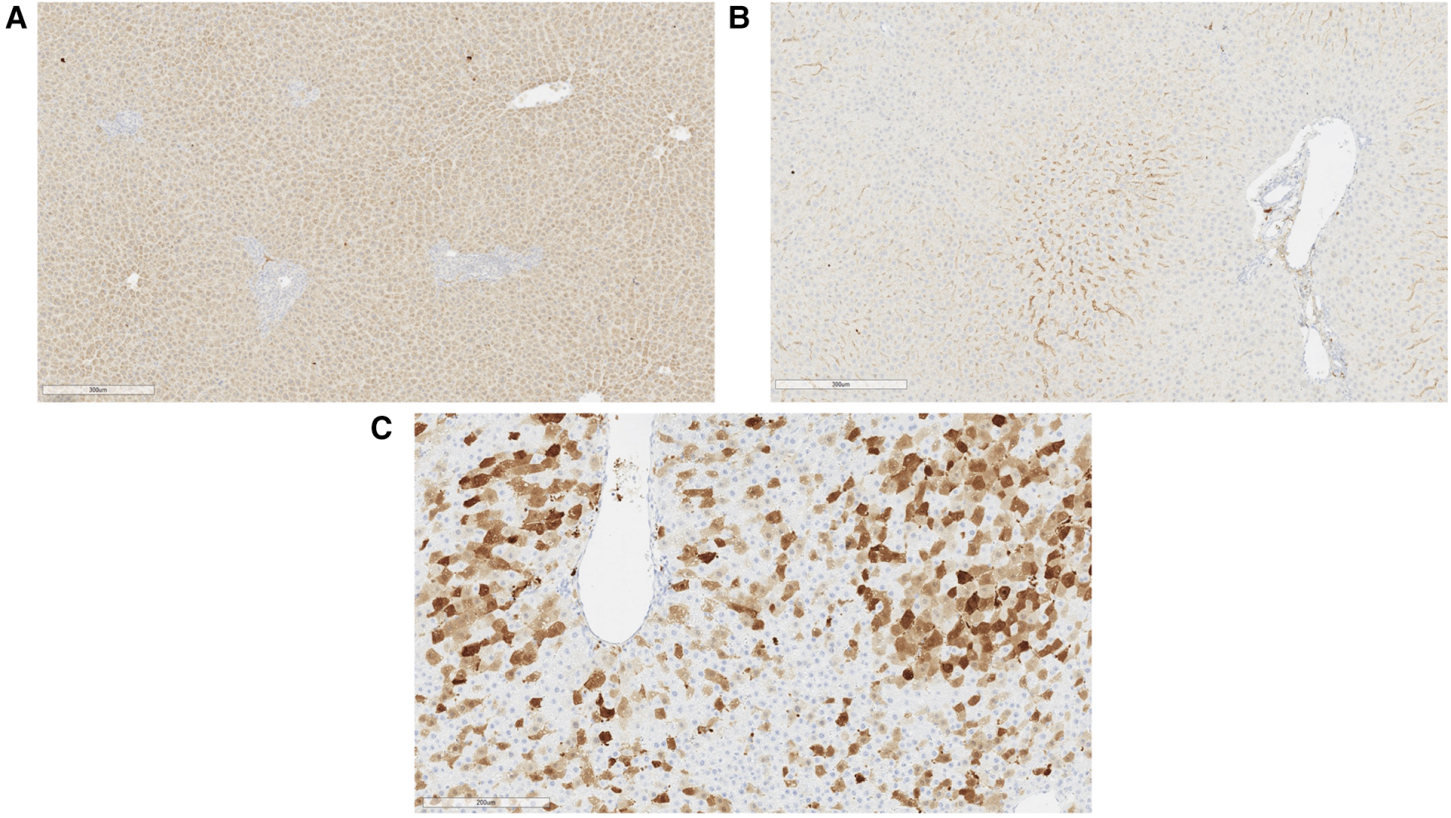
Representative immunohistochemical images of liver tissue at experimental endpoint. (**A**) Generalized luciferase expression in rat hepatocytes was observed at sacrifice with sparing near the portal structure (right). This animal received a liver allograft treated with intermediate dose AAV9-Luc and was sacrificed on posttransplant day 56. (**B**) Luciferase immunohistochemical image of liver from a control animal on posttransplant day 56. (**C**) Significant GFP expression was noted in hepatocytes on posttransplant day 5 when self-complementary AAV9 vector was utilized. This animal received 8e10 vg sc-AAV9-GFP and was sacrificed on posttransplant day 5.

At the 2-month experimental endpoint, tissues from the heart, lung, liver, spleen, and kidney were collected for qPCR and the measurement of tissue luciferase activity *in vitro*. Tissues from animals that received high-dose AAV9-Luc were not subjected to further analysis since it is unclear whether the surviving animals received the full intended dose of recombinant AAV9. Luciferase activity in the liver graft was significantly higher among animals that received the intermediate dose in comparison to the low-dose group (control group: 2.5e3 RLU/g, low-dose group: 8.6e3 RLU/g, intermediate dose group: 2.6e5 RLU/g, *P* = 0.0048) (Figure 4A). The same trend was observed in the presence of viral genomes in graft tissue by qPCR (control group: 1.1e2 vg/µg DNA, low-dose group: 2.5e3 vg/µg DNA, intermediate dose group: 1.1e4 vg/µg DNA, *P* = 0.0048) (Figure 4B). Lastly, transgene expression at the experimental endpoint was confirmed using immunohistochemistry (Figure 5A), which demonstrated transgene staining predominantly in hepatocytes.

A separate set of syngeneic liver transplants was performed using GFP. Sc-AAV9-GFP was delivered to the donor graft as described above. Unlike the single-stranded AAV9-Luc used in earlier experiments, the sc-AAV9-GFP used has a self-complementary design, which results in more rapid transcription upon delivery. Here, we observed extensive transgene expression throughout the liver parenchyma by posttransplant day 5 (Figure 5B).

### No off-target expression of transgene is observed

A potential concern with the intravascular delivery of recombinant AAV9 is the possibility of vector washout and subsequent off-target expression of the transgene. Although our experiments were limited by a relatively small sample size, no obvious transgene expression was detected in the lung, heart, spleen, and kidneys based on *in vivo* bioluminescence imaging (Figure 2A,B), tissue luciferase assay (Figure 4A), or qPCR (data not shown).

## Discussion

This is the first study to demonstrate successful AAV9-mediated gene delivery to liver grafts during static cold storage. Transgene expression was detected as early as 7 days posttransplant using a single-stranded AAV9 construct and 5 days posttransplant using a self-complementary AAV9 construct. The expression of the reporter gene remained detectable at 2 months posttransplant, suggesting a durable response. Following delivery in this manner, no obvious off-target transgene expression was observed.

The long-term gene expression observed in this study highlights the potential of AAV9-mediated gene delivery to achieve durable graft modification. In comparison, prior studies using adenoviral mediated gene delivery were characterized by a rapid onset and offset of gene expression. Benigni et al. delivered an adenoviral vector to kidney grafts and noted β-gal expression as early as 2-day posttransplant (3). Olthoff et al. delivered recombinant adenovirus encoding CTLA4-Ig to the donor liver during static cold storage. In their study, messenger RNA for CTLA4-Ig was detected at high levels within the first 4 days but rapidly declined to undetectable levels by day 14. Serum levels of CTLA4-Ig were high within the first 10-day posttransplant, but very few animals had demonstrable levels of CTLA4-Ig beyond posttransplant day 20 (2). These findings are consistent with the limited duration of transgene expression seen with adenovirus in non-transplant settings (9).

In this study, we chose to deliver AAV9 during static cold storage since this remains the simplest and most common method of organ preservation across all solid organs. The successful delivery of transgenes to solid organ grafts under conditions of SCS has previous precedent, with a prior demonstration of effective adenoviral mediated gene delivery at 4°C (6, 10). This approach has also been demonstrated using AAV vectors. Yang et al. first described the use of AAV-mediated gene delivery during SCS in a rat liver transplant model in 2003 (11). In that study, the authors demonstrated successful expression of transgene CTLA4-Ig in the transplanted liver for up to 180 days. However, it is unclear whether the immunosuppressive effect of CTLA4-Ig impacted the duration of transgene expression. In addition, the authors did not evaluate the potential off-target effect of AAV.

The recent development of organ perfusion systems presents an intriguing alternative for gene delivery, although with an attendant increase in complexity. In a recent report, Bonaccorsi-Riani et al. delivered AAV8 encoding GFP to rat liver grafts during hypothermic oxygenated machine perfusion and evaluated the tissue using immunohistochemistry 24 h after transplantation. Although 24 h is considered early for AAV-mediated transgene expression, the authors demonstrated evidence of GFP expression in hepatocytes (12). Normothermic machine perfusion approaches are particularly attractive for therapeutic delivery due to the maintenance of metabolic activity of the graft during perfusion. Using this approach, Yeung et al. delivered recombinant adenovirus expressing human IL-10 to porcine lung allografts and were able to detect high levels of human IL-10 expression by the end of a 12-h *ex vivo* lung perfusion (13). Further studies are necessary to determine the optimal method of AAV delivery, weighing the simplicity and wide applicability of SCS vs. the potential for improved uptake using machine perfusion approaches.

In contrast to our recent experience with AAV9-mediated gene delivery to lung allografts (14), in this study, we observed an increased mortality among animals that received high doses of AAV9 (4e11 vg). These deaths occurred within the first few days posttransplant, and the mechanisms remain unclear. Recombinant AAV has been postulated to activate the host innate and adaptive immune system in recent years, especially at higher doses (15). Hepatotoxicity specifically has been reported after the *in vivo* administration of large doses of recombinant AAV in animal models and clinically (16, 17), especially given the strong tropism toward the liver. This aspect deserves further exploration in a large animal model to clarify the dose–response relationship for transplant applications.

There are several limitations of our study that deserve mention. First, this study is limited by a relatively small sample size. In addition, while the rat liver transplant model has been well-validated in the literature, these findings require confirmation in large animal models. Finally, we only explored gene delivery in the context of static cold storage organ preservation, and future studies incorporating delivery during *ex vivo* machine perfusion will allow a comparison of these modes of organ preservation.

In conclusion, we have demonstrated the feasibility of robust and durable transgene expression in liver grafts using AAV9-mediated gene delivery. This approach has the potential for rapid clinical translation in transplantation, as AAV9-based therapeutics have already achieved FDA approval for non-transplant indications. Although declining levels of transgene expression can occur with cell division, sustained transgene expression was observed in our study, similar to that documented in clinical trials (18, 19). As such, AAV-mediated gene therapy may prove ideal for long-term graft modification, specifically for immunological targets to control the local immune response. Proof-of-concept studies in large animal models and involving specific immunologic targets will be the next step in applying the recombinant AAV technology in transplantation.

## Data Availability

The raw data supporting the conclusions of this article will be made available by the authors, without undue reservation.

## Supplementary material

Supplementary Figure S1 In vivo Bioluminescence Images of all transplant recipients at posttransplant 7, 14, 28 and 56 days, across different treatment groups. Four different scales were used in this figure. Images circled in the green boxes has a scale of 1.5 – 5 × 10^5^ p/s/cm^2^/sr. Image circled in the blue box, 1.5 – 10 × 10^5^ p/s/cm^2^/sr. Images circled in the purple boxes, 5 – 10 × 10^5^ p/s/cm^2^/sr. Images circled in the red boxes, 1 – 10 × 10^6^ p/s/cm^2^/sr.
