# Supplementary figures and images for: AAV9-mediated gene delivery to liver grafts during static cold storage in a rat liver transplant model
Source: Front Transplant. 2023 May 30;2:1171272. doi: 10.3389/frtra.2023.1171272 (PMC11235296; doi:10.3389/frtra.2023.1171272)

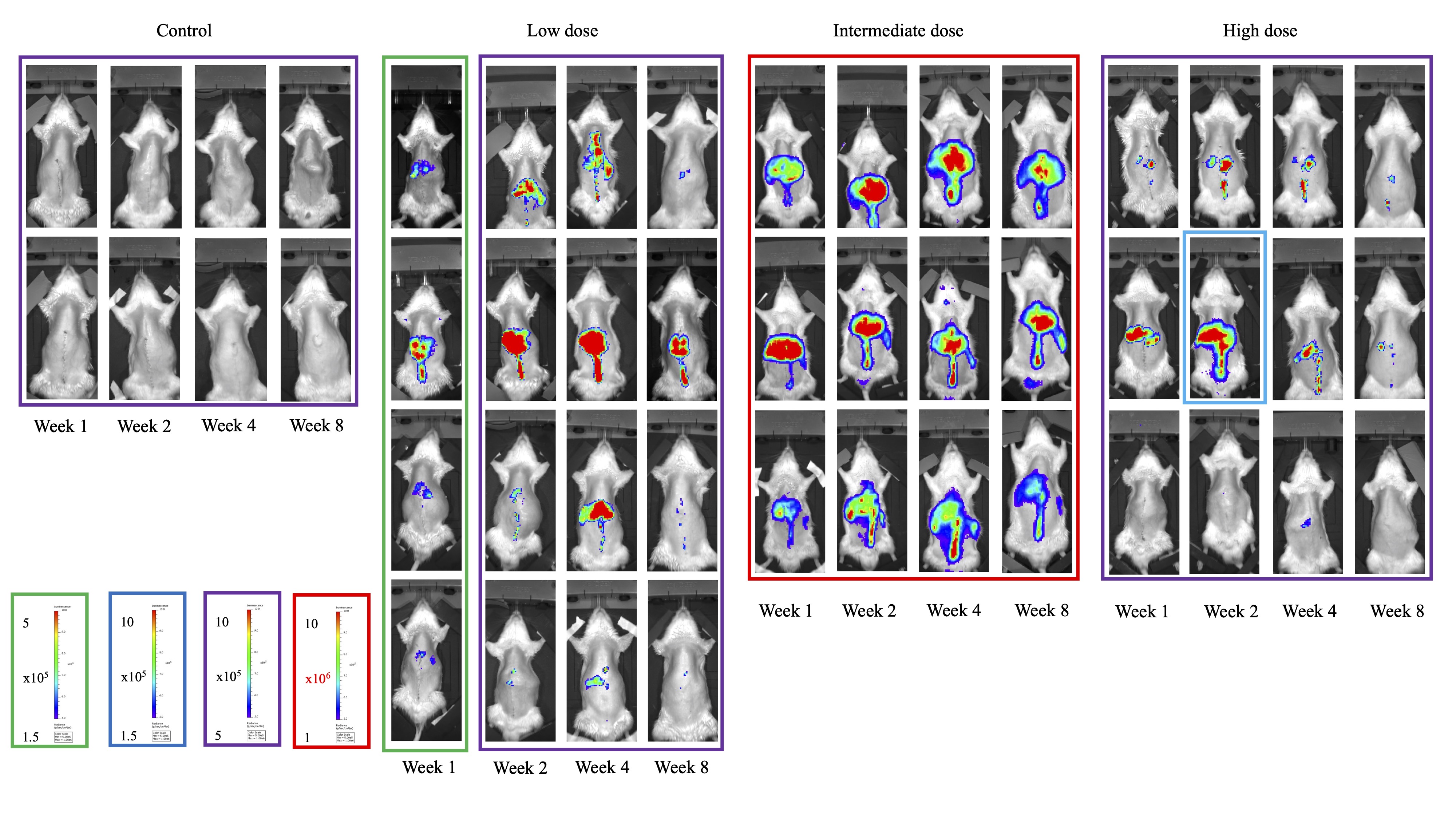

Supplement: Supplementary Figure S1 — In vivo Bioluminescence Images of all transplant recipients at posttransplant 7, 14, 28 and 56 days, across different treatment groups. Four different scales were used in this figure. Images circled in the green boxes has a scale of 1.5 – 5 × 10^5^ p/s/cm2/sr. Image circled in the blue box, 1.5 – 10 × 105 p/s/cm2/sr. Images circled in the purple boxes, 5 – 10 × 105 p/s/cm2/sr. Images circled in the red boxes, 1 – 10 × 106 p/s/cm2/sr. [file Image1.jpeg]
